# Inferences made from mineral elements characterisation of anaerobic digestion substrates: preliminary results from avocado oil processing byproducts

**DOI:** 10.1007/s10532-025-10125-5

**Published:** 2025-04-10

**Authors:** Charles Rashama, Grace N. Ijoma, Tonderayi S. Matambo, Christian Riann

**Affiliations:** 1 Department of Life and Consumer Sciences, College of Agriculture & Environmental Science, University of South Africa, Cnr Pioneer and Christian De Wet Roads, Private Bag X6, Florida, 1710 South Africa; 2 Centre of Competency for Environmental Biotechnology, College of Agriculture & Environmental Science, University of South Africa, Cnr Pioneer and Christian De Wet Roads, Private Bag X6, Florida, 1710 South Africa; 3 Department of Environmental Sciences, College of Agriculture & Environmental Science, University of South Africa, Cnr Pioneer and Christian De Wet Roads, Private Bag X6, Florida, 1710 South Africa

**Keywords:** Avocado, Biogas, Inhibition, Minerals, Nutrients, Microorganisms

## Abstract

The mineral element levels in avocado oil processing byproducts were evaluated to make inferences about these byproducts’ biodegradability behaviour. The mineral assays were determined using ICP-OES methods. Literature was also reviewed to understand how different mineral elements detected in substrates could impact biogas production under anaerobic digestion (AD) conditions. It was noted that there is no consensus among scholars on absolute mineral requirement levels for optimal digester performance since digesters use different substrates operating under varying conditions in most cases. When mineral elements are below certain values, the digester operates sub-optimally. When certain mineral levels are exceeded in digesters, the minerals become toxic to the AD microorganisms and destabilise the biogas digester. Only Zn levels across all avocado oil processing byproducts and K levels in cold-pressed decanter wastewater (CDW) exceeded the toxic level limits pegged at 1 mg/L and 3000 mg/L respectively. These may therefore need corrective action to avoid potential inhibition of the AD process. Mineral assays that seemed adequate and required no supplementation or detoxification were K, P, Fe, Cu, Se and Cr across the studied byproducts. All the other elements (excluding the Hg, As and Cd which are toxic) may require supplementation for optimal digester performances when using avocado oil processing byproducts as biogas digester substrates. However, these inferences may be affected by issues of mineral bioavailability which is a substrate-dependent phenomenon. Mercury cadmium and arsenic are naturally toxic to microorganisms and must be removed or detoxified if detected in substrates. The current study indicates the significance of mineral elements’ characterisation in explaining and potentially guiding the optimisation of substrate biodegradation to biogas.

## Introduction

Among many waste management options available to handle avocado oil processing byproducts, the inclusion of anaerobic digestion (AD) as part of a biorefinery has been reported to be the most viable route from a sustainability point of view (Garcia-Vallejo et al. 2023; Sandoval-Contreras et al. 2023). The biodegradation of avocado oil processing byproducts to produce biogas as an energy carrier gas is therefore gaining popularity in research (Sandoval-Contreras et al. 2023; Rashama et al. 2020) and at the commercial level (Olivado 2025). The use of avocado oil processing waste biomass in AD process addresses pollution, energy and agricultural fertiliser issues while freeing up productive land occupied by landfills and lagoon ponds in current methods of managing these wastes. Therefore, research that promotes AD use on avocado oil processing byproducts valorization is welcome for sustainable development. Rashama et al. (2022) pointed out low biodegradabilities ranging from 10–29% in avocado oil processing byproducts attributing these low values to phytochemicals occurrence in these substrates. Phytochemicals exhibit antimicrobial characteristics which explains the poor performance of the biogas digesters. Vintila et al. 2019, also reported sub-optimal biomethane potentials from avocado seeds until they had to include the expensive steam and cellulose pretreatments to the avocado seeds. Their biomethane potential (BMP) result without the pretreatment was 106 mL/gVS which equates to a 37% material biodegradability, also way below the 50% mark reported for most substrates. However, a lot more work needs to be carried out to optimize the biodegradation of these substrates and encourage their valorization through anaerobic digestion. In addition to phytochemicals, other substrate characteristics such as long chain fatty acids content, lignin content, inorganic minerals content, etc. could also contribute to the cited low biodegradabilities. As a standard procedure, Researchers normally perform proximate and ultimate characterisation of substrates to understand substrate behaviour in AD performance (Matheri et al. 2019). Recently, a study by Rashama et al. (2021) outlined the importance of phytochemicals in ruminant and biogas digester microbial dynamics. This research recommended that the phytochemical characterisation of substrates should also be incorporated in standard substrate testing for any previously untested biodigester substrates of plant/crop origin.

The determination of inorganic mineral elements in substrates is also understudied. These mineral elements are reported to inhibit or promote microbial processes (Kadam et al. 2022) in AD. However, other researchers omit this characterisation step for reasons not immediately known to us and other practitioners (Chala et al. 2018; Du et al. 2021). Substrate mineral element profiles can be studied to assist in the optimisation of biodigester substrate selections for co-digestion decisions (Bozym et al. 2015). Mineral elements are also investigated as a precursor to understanding the AD digestate’s properties and making decisions regarding digestate use as a biofertiliser (Stefan et al. 2021). For biofertiliser application, metal levels in the slurry must not exceed certain limits (Lane et al. 2024). Generally, the role and effects of elements in digesters are poorly understood by many AD practitioners (Ezebuiro and Korner 2016) although a few scholars have indicated that mineral elements could induce both inhibition or biostimulation of microbial processes depending on species and concentrations involved (Myszograj et al. 2018). The reasons behind the omission of substrate mineral elements characterization in some studies need to be established. The compulsory inclusion of substrate pH, VFA, total Kjeldahl nitrogen, ammonium, and alkalinity analysis in standard BMP tests on the ground that these variables help to explain inhibition in digesters, also justifies the compulsory mineral elements characterisation as well in substrate BMP tests (Holliger et al. 2016). Avocado oil processing byproducts as new AD substrates on the market have not been adequately studied especially on the aspect of mineral element effects on substrate biodegradability. This study’s objective is to determine the concentrations of heavy and light metal elements in avocado oil processing byproducts and then couple this information with literature review findings to infer the AD behaviour of these byproducts. These inferences help shape future research and guide the development of optimal AD operational strategies when working with the studied substrates. The byproduct samples were available in one season only. Therefore, no biological replicates based on different seasons could be used in the research design. With this challenge, limited experimental analysis was performed, rendering the results and discussion only preliminary as captured in the title.

## Materials and methods

### Literature survey

A literature search using keywords such as macro and microelements, mineral and trace nutrients, inorganic elements, etc. in biodigesters or anaerobic digestion was conducted on Google Scholar, Scopus and ScienceDirect databases. Some researchers classify mineral elements into macro and microelements, with others preferring to name these trace elements or nutrients instead. Information about inorganic elements’ effects on digesters, the acceptable ranges and other important issues were populated for subsequent use in inferential studies involving avocado oil processing byproducts’ mineral element analysis.

### Materials sourcing and handling

Westfalia Fruit (Pvt) Ltd with operations in Tzaneen and Howick (South Africa) provided the avocado oil processing byproducts. Two technologies are employed in the company’s operations. The hot process technology was used at the Tzaneen factory where whole fruit avocados are crushed for oil production. The cold process technology practised at Howick involves the removal of fruit peel and stone before crushing. After crushing, the pulp is malaxed at 55–60 °C for the hot process and 45–48 °C for the cold process. Oil is later squeezed out of the malaxed pulp using centrifugal separators. The hot process generates pomace (HDP) and wastewater (HDW) while the cold process generates seeds (AK), peels (AS), pomace (CDP) and wastewater (CDW). Samples were collected fresh during fruit processing and then transported in cooler boxes (4 °C) to the University of South Africa, Science campus laboratories. All samples were air dried until reading constant weight within 5 days after collection. The dry samples were then profiled for macro and microelements at Agricultural Research Council (ARC) laboratories in the Institute of Soil, Water and Climate unit. All chemicals sourced from Sigma Aldrich (South Africa) were of analytical grade.

### Substrate mineral and trace element analysis

The air-dried samples were milled and weighed (1 g) and each sample was digested in two ways following standard guidelines (Sailor et al. 2020; Sleimi et al. 2022). For mineral element analysis, the 1 g sample was digested at 180 °C using 7 ml of conc nitric acid and 3 ml of conc perchloric acid while adding a few drops of hydrogen peroxide. Upon cooling, 10 ml of 1:1 hydrochloric acid was added to this mixture. Contents were topped up to 100 ml in a conical flask using distilled water. The resulting digest solution was ready for mineral elements analysis using Inductively Coupled Plasma—Optical Emission Spectrometry (ICP-EOS). For trace element analysis more concentrated solutions are required so a separate sample digestion was done in the same way as for mineral elements but with sample quantity increased to 5 g, temperature reduced to 120 °C and final volume reduced to 50 ml. Twelve (12) mineral elements and nine (9) trace elements were determined from the digest solutions using an Agilent 725 ICP-EOS Simultaneous instrument. Phosphorous in the avocado oil processing byproducts was determined using colourimetric methods outlined by Wieczorek et al. (2022). Briefly, this involved drying samples, preparing digests of each byproduct using the suggested solvents then measuring the absorbency of the digests and concentration read from a calibration curve developed using standards. The protocol which incorporates pH adjustment was followed for phosphorous determination. Ammonium nitrogen determination was conducted through spectroscopic methods whereby the pulverised sample was soaked in deionised water to dissolve the available ammonium. Finally, the ammonium concentration was quantified using a Berthelot method on the TRAACS 800TM AutoAnalyzer (Shariat-Rad and Qanei 2022).

### Data analysis

Statistical analysis and reporting of experimental results were performed following guidelines stated by Lang et al. (2015). This was an exploratory study aimed at identifying the difference in the specific mineral element assay across samples. The assays follow a continuous scale. Each of the six samples was independent but received the same treatment for analysis of a specific mineral element. There were six (6) groups and no control samples or control treatments. These conditions were adequate for applying a one-way ANOVA test. The ANOVA test was undertaken to check for significant differences in the mineral element assays across samples (groups). The biomass type is the only factor expected to cause variations in mineral element levels in this study. A Microsoft Excel computer program was used to perform the ANOVA test at 95% confidence interval (α = 0.05). Mineral element assay results for each element on each of the six (6) different biomass samples that were analysed from each of the two sites were combined for the two sites to get an average value that represented both sites and a sample name. Variances and standard deviations in the results were calculated for each mineral element.

## Results and discussion

It was deduced from the available published literature that there are various reasons why some researchers in AD studies conduct mineral elemental analysis while others do not. Some of these reasons and their implications are noted in Table 1 with the supporting literature cited also.

**Table 1:** Reasons for inclusion or non-inclusion of mineral element characterization in AD studies

Reason	Explanation and implication
There is no agreement among researchers regarding optimal metal concentrations for efficient anaerobic digestion	Requirements recommended by different authors vary from one study to the other (Thanh et al. 2016; Schmidt et al. 2018). These variations tend to justify why other authors choose not to analyse minerals as they cannot make informed deductions from those results
There is no universal standard for biomethane potential (BMP) evaluation protocols	For convenience, authors choose protocols that address their key research questions without looking at what other protocols emphasise. Efforts to standardise substrate biomethanation evaluations are still in progress (Holliger et al. 2016, 2021)
Cost (time and money) and equipment or expertise availability	Analysis of mineral element assays and bioavalabilities is important but it takes time to run the sequential extraction procedures. These procedures use different costly extractants (Choong et al. 2016). Other researchers can’t afford this. Then ultimately, they focus on other characterisation that can be conducted with available resources
Prior knowledge about the substrate from independent and other studies	If a researcher wants to investigate certain aspects of a substrate and that substrate has been previously characterised for metals elsewhere and found to be within acceptable digester ranges, rather than repeating metal analysis, the researchers go straight into investigating issues of concern relating directly to their research and leave out the already known metal profiling information
Inconsistent nomenclature	There is a proliferation of classification terminologies such as mineral elements, macronutrients, trace elements, inorganic elements, and micronutrients without a consistent definition of these by researchers. To escape, these confusions, some researchers then opt not to characterise or include substrate minerals assays in their studies (Romero-Güiza et al. 2016; Roussel et al. 2018)

**Fig. 1:**
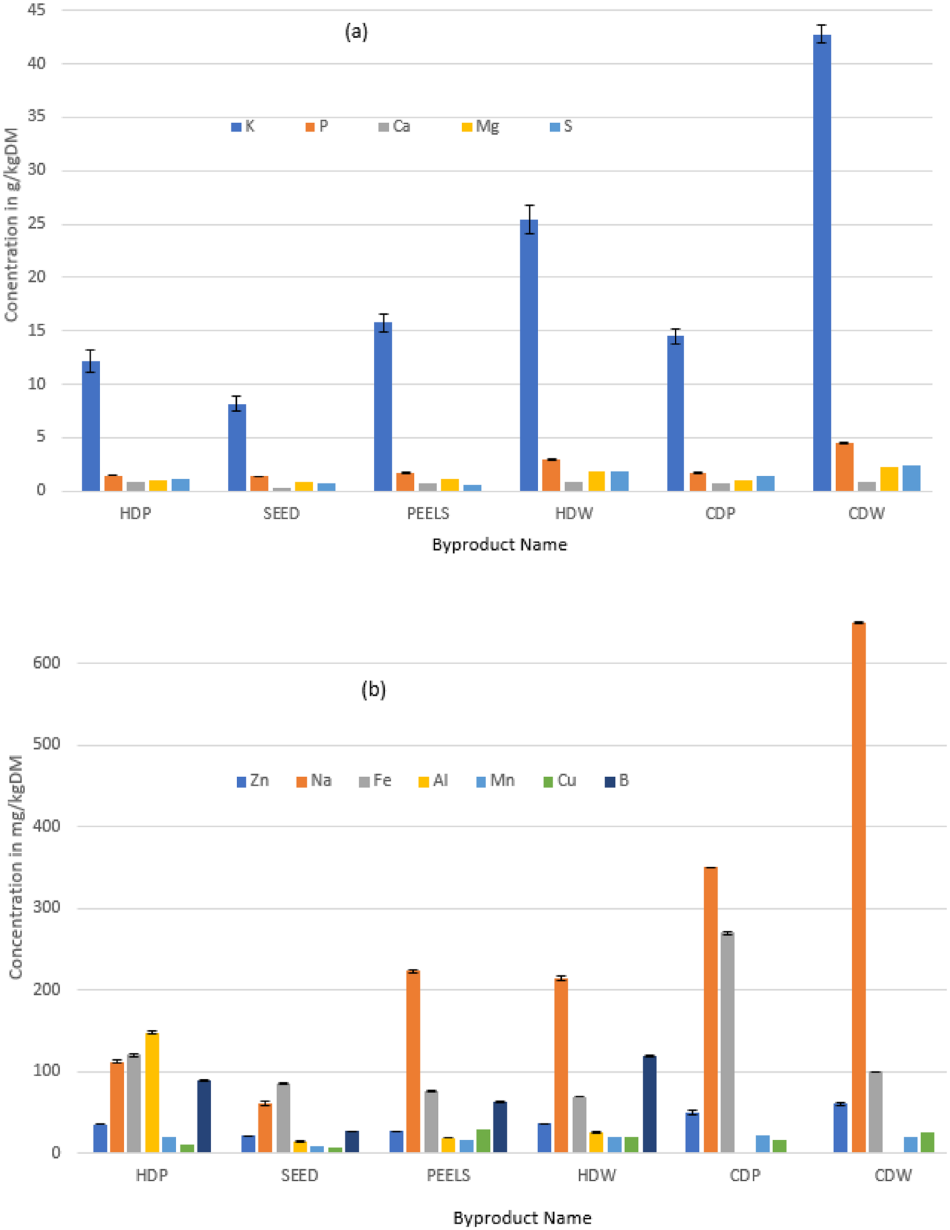

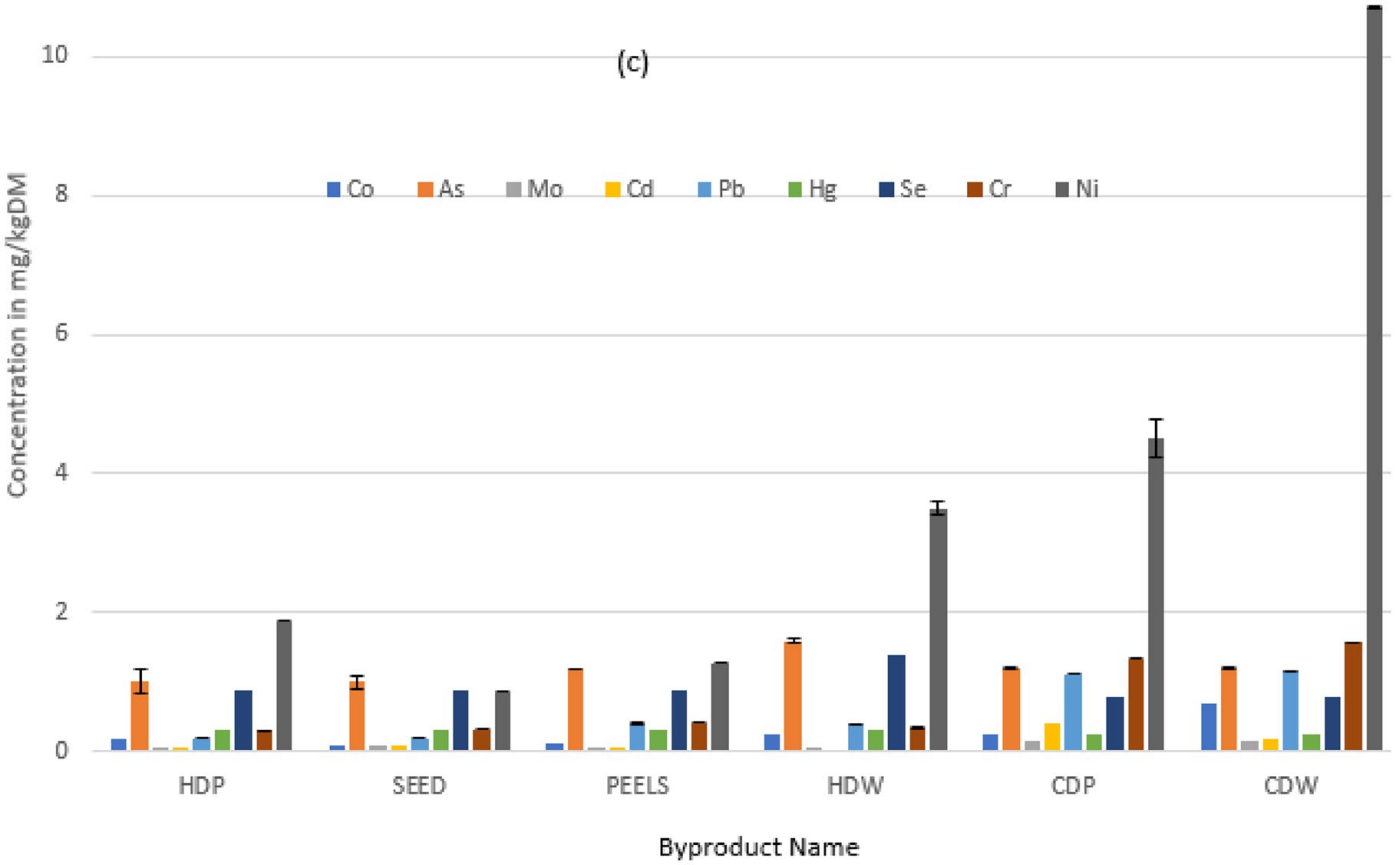
Macro and microelement composition of substrates **a** High **b** Medium **c** Low concentrations. Results are presented as means with standard deviation representing error bars representing the standard deviation. (P-value = 0.828; α = 0.05)

**Fig. 2:**
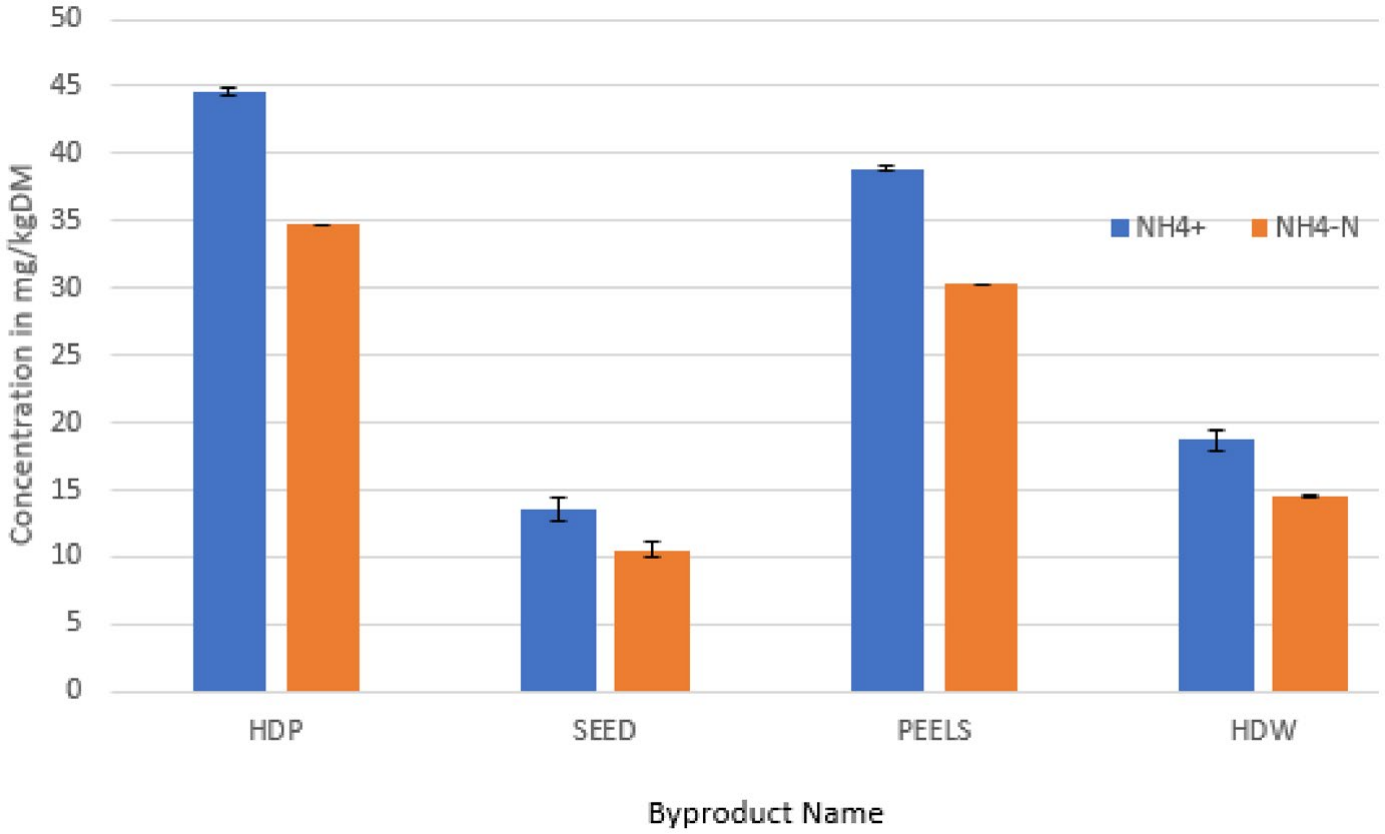
Ammonium and ammoniacal nitrogen concentration in byproducts. Results are presented as means with error bars representing the standard deviation

Metal requirements in digesters and AD behaviour inferences made from avocado oil processing byproducts mineral analysis are summarised in Table 2. Mineral element assay results of avocado oil processing byproducts are displayed in Fig. 1 while Fig. 2 displays nitrogen levels in avocado oil processing byproducts. The studied byproducts had eleven (11) mineral elements falling within acceptable ranges for optimal AD although Mo, Se, Ni and Cr were on the lower end of these ranges implying that their supplementation may be necessary for digesters using these substrates. Eight (8) mineral element assays across the avocado byproducts were below acceptable ranges for optimal biodigester performance. These elements require supplementation in digesters. Potassium is within range in five byproducts except in CDW where it is slightly higher than the toxicity level of K in digesters. Across all byproducts, P concentration was exceeded by more than 1000% of the max requirement for digesters although the toxicity level of this element in digesters could not be established from the literature consulted. There are high chances of inhibition or toxicity from P across all byproducts because of this excessively high level above the required amounts. Zinc also assayed above the toxicity level across all substrates and this calls for implementation of detoxification strategies for this element. The mineral element levels and potential toxicities of each of these elements during AD of these byproducts are discussed in Table 2. None of the heavy toxic metals were detected in high enough concentrations across the avocado oil processing byproducts to induce microbial toxicity. However, attention must be given to Hg and Cd which are detected in the range of 0.002 to 0.032 mg/L across avocado oil processing byproducts and are toxic to microbes despite their low concentrations. These metals including As pose health threats to humans as well if they may potentially enter the food chain through biogas digestate used as biofertilizer in human-consumed crops (Sturmer et al. 2020). Lower levels of certain heavy metals can be tolerated and sometimes certain microorganisms at these low metal concentrations can biotransform these heavy metals reducing their toxicities. Some metals (Fe, Zn, Ni, Co), when existing in acceptable concentrations have been reported to induce microbial growth and biostimulation for certain species in AD consortia which ultimately improve biogas yields (Borowski et al. 2023). A detailed analysis of specific mineral elements’ roles in biogas digesters has been reported by Roussel et al. (2018) and Myszograj et al. (2018). Readers are therefore directed to these resources for a deeper understanding of this topic as reproducing the contents of these studies in the current study is out of scope. Heavy metals have been reported to complex with thiols and disrupt enzyme activities hence AD microbial activities (Xin et al 2019). Reported heavy metal toxicities in digesters towards Total Solids (TS) reduction, total chemical oxygen demand (TCOD) reduction, biogas yield and methane content were ranked in ascending order as Pb < Cr ≈Zn < Cu, Pb < Zn < Cr < Cu, Cu > Zn ≈Cr > Pb, and Pb < Cr < Zn < Cu respectively (Al bkoor Alrawashdeh 2022). To indicate the importance of certain metals to microorganisms, methanogens were found to contain metals in their bodies in the order Fe >  > Zn > Ni > Cu ≈ Co ≈ Mo > Mn implying that these metals play a crucial role in these microbes (Choong et al. 2016). Though beneficial to microbial cell functions, the inhibitory concentrations for these metals are generally low calling for digester operators to exercise vigilance in monitoring these metals in digesters. Agro-processing residues usually lack the required minerals for optimal digestion as opposed to industrial-based AD substrates (González-Suárez et al 2016). The mineral elements characterization results for avocado oil processing byproducts indicate that generally the risks of toxicity/inhibition of microbial activity in biogas digesters using these byproducts as substrates are low. Only zinc and phosphorous seemed to present a potential challenge in this regard across all byproducts. The high assay of zinc results reported in our study corroborate with the findings reported by Newett et al. (2018) which showed that avocado plants are a zinc-demanding crop for good fruition. Though digester mineral element requirements reported in Table 2 are based on different substrates and operating conditions, they give an idea of the general ranges so far identified from scholarly AD works. This allows anaerobic digestion inferences based on mineral element analysis of the substrates being considered in this study to be made.

**Table 2:** Digester mineral requirements/limits and inferences from studied substrates’ characterisation

Metal	Low needs	High needs	Toxic levels	Avocado oil byproducts digestion inferences and strategies (Assume 8% TS in digester slurries) (Rashama et al. 2019)	References
	mg/L				
K	–	1423.8	3000	All byproducts gave digester slurry K concentrations in the range of 656 mg/L (seed) to 2035 mg/L (HDW) which is adequate and below toxic levels except for 3424 mg/L (CDW) which is slightly above the toxic limit	Bozym et al. (2015), Wu et al. (2016)
P	0.1	1	–	Lowest P in seeds at 109 mg/L while the highest is 359 mg/L (CDW). P is more than adequate in all substrates and toxicity levels are not known although too high a concentration is claimed to trigger struvite precipitation which takes up other metals when it happens and may probably then induce deficiencies in other trace elements	Roussel et al (2019)
Ca	100	1035	> 2500	The lowest Ca in peel slurry is 55 mg/L while the highest value is 68 mg/L (CDW). Ca needs to be supplemented in all substrates. There were no threats of Ca toxicity presented by these substrates	Bozym et al. (2015), Romero-Güiza et al. (2016)
Mg	360	4800	> 2400	Mg concentration in digesters was lowest at 69 mg/L (seeds) and highest at 184 mg/L (CDW). Mg supplementation to meet minimum requirements is necessary in all substrates	Bozym et al. (2015)
Zn	0.15	0.32	1	The lowest Zn levels in the slurry are 1.7 mg/L (seeds) while the highest value is 4.8 mg/L (CDW). All byproducts assay Zn concentrations above the toxicity limit. Strategies of detoxification (co-digestion, removal, chelation) may need to be explored	Bozym et al. (2015; Schmidt et al. 2018)
Na	100	350	6000–30000	The lowest Na assay in the byproducts gives a slurry concentration of 4.9 mg/L (seeds) and the highest value is 52 mg/L (CDW). These values are way below Na toxicity levels though information on requirements for optimal substrate degradation to biogas are also unknown	Bozym et al. (2015), Romero-Güiza et al. (2016)
Fe	0.43	400	10	Within the acceptable range, the lowest Fe concentration in digester slurry is 5.5 mg/L (HDW) while the highest value is 9.6 mg/L (HDP). Potential inhibition may be observed in CDP where slurries will have Fe concentrations of 22 mg/L	Thanh et al. (2016), Schmidt et al. (2018)
Al	–	–	1000	All substrates produce slurries with Al assays (1.1–11.8 mg/L) below the toxic level of 1000 mg/L. Though CDP and CDW could not be characterised, it is also expected that these two will also be safe from reaching toxic levels since they are derived from the same fruits where the other byproducts were way far below the toxic limit	Myszograj et al. (2018)
Mn	8	120	–	The lowest Mn concentration in digester slurries is 0.7 mg/L (seeds) and the highest value is 1.6 mg/L (CDW). This means Mn needs to be supplemented in digesters that use avocado oil processing byproducts as substrates	Schmidt et al. (2018)
Cu	0.06	0.512	3.2	Slurry concentration of Cu was lowest 0.5 mg/L (seeds) and highest 2.3 mg/L (peels). These values were more than adequate for optimal digestion and are still below toxicity levels	Bozym et al. (2015)
B	–	–	–	No informative AD studies addressing digester requirements for this element were found. The element has been found in methanogens and is claimed to be a cofactor in enzymes. In the studied byproducts, boron assays were lowest at 26 mg/kgDM (seeds) and highest at 119 mg/kgDM (HDW)	Choong et al. (2018)
Co	0.032	8	–	Cobalt (Co) in the byproducts gives a slurry concentration of 0.008 mg/L (seeds) and the highest value is 0.05 mg/L (CDW). Only CDW is capable of slightly exceeding the minimum Co requirements of digesters. Cobalt supplementation is therefore recommended for the other substrates but not in the case of CDW	Thanh et al. (2016), Schmidt et al. (2018)
As	–	–	–	Sludges from byproducts measured low As concentration of 1.0 mg/kgDM and highest 1.6 mg/kgDM. These values are below the ceiling values permissible in bio fertilisers (< 40 mg/kgDM) implying that digestates from these byproducts can be safely used as biofertilizer	Sturmer et al. (2020)
Mo	0.0024	1.28	–	Lowest Mo assay in the byproducts gives slurry concentration of 0.004 mg/L (peels, HDW and HDP) and highest value is 0.012 mg/L (CDW and CDP). Though these values meet minimal requirements, they are way below the max requirements stated in literature. Supplementation of Mo in all the byproducts may be necessary to meet high demands	Schmidt et al. (2018)
Cd	–	1.6	14.4	All by-product substrates result in low Cd assays in digester slurries between 0.0024 mg/L (HDW) and the highest value is 0.032 mg/L (CDP). These values are way below Cd toxicity levels and below Cd requirements for optimal substrate degradation to biogas. All byproducts require supplementation but take cognizance of not exceeding the safe Cd limit of < 0.3 mg/kgDM for biofertilizer use	Bozym et al. (2015), Romero-Güiza et al. (2016), Sturmer et al. (2020)
Pb	0.02	200	340	Low Pb assays in digester slurries between 0.01 mg/L (HDW) and the highest value is 0.09 mg/L (CDP) across byproducts are inadequate to support biodigestion or cause toxicity in digesters. Supplementation may be necessary	Bozym et al. (2015), Matheri et al. (2016)
Hg	–	–	–	Sludges from byproducts assay Hg concentrations lowest 0.25 mg/kgDM and highest 0.3 mg/kgDM. These values are below the ceiling values permissible in biofertilisers (< 1.0 mg/kgDM) implying that digestates from these byproducts can be safely used as biofertilizers	Sturmer et al. (2020)
Se	0.004	0.8	–	Selenium (Se) assays in digester slurries across byproducts are lowest at 0.06 mg/L (CDW) and highest at 0.112 mg/L (HDW). These values are within the recommended range for Se requirements in digesters though on the low end of the range. Supplementation may therefore be necessary	Thanh et al. (2016), Schmidt et al. (2018)
Cr	0.005	50	130	All by-product substrates reported low Cr assays iranging between 0.025 mg/L (HDP) and highest value is 0.13 mg/L (CDW). These values are in the recommended range of Cr requirements though on the lower end. There is no risk of toxicity and supplementation is necessary to reach optimal substrate degradation to biogas	Bozym et al. (2015)
Ni	0.029	8	10	Nickel in byproducts will give digester slurries concentrations ranging lowest 0.07 mg/L(seeds) and highest 0.8 mg/L (CDW). These values are way below Ni toxicity levels though they meet minimum requirements in digesters. Supplementation is required to meet maximum demands	Bozym et al. (2015), Thanh et al. (2016)

Ammonium and ammonium nitrogen analysis is also critical in AD studies. These parameters should be checked alongside mineral element analysis for the same reasons as those outlined in the latter’s case. Methanogens are more sensitive to ammonium and ammoniacal nitrogen than the other three distinct microbial groups found in digesters. However toxicity on different classes of the digester microbiota from NH_4_^+^ ions has been reported for concentrations ranging 1500–7000 mg/L (Jiang et al. 2019). In many cases ammonia inhibition is compounded by VFA inhibition which kicks in when the rate of acidogenesis overtakes that of methanogenesis. Yan et al. (2019) noted that during AD studies with organic fraction of municipal wastes, when NH_4_^+^ concentration reached 8000 mg/L even with acclimatization, the VFA levels also rose sharply. These digester conditions reduced the methane production rate which was a reflection of an imbalanced methanogen/acidogen ratios. Figure 2 depicts the NH_4_^+^ and NH_4_N results for four of the avocado oil processing byproducts. Assays for the other two byproducts could not be completed because of equipment failure. The depicted results translate to a maximum of 3.6 mg/L ammonium ions in digesters slurry for HDP with the other substrates registering even less concentrations. This implies that there is no risk of ammonium toxicity from these substrates. Allowable free ammonia requirements in digesters have been suggested to range widely between 50 and 1500 mg/L to avoid toxicity to methanogens (Mutegoa et al. 2020). This indicates the need for ammonium supplementation in digesting avocado oil processing byproducts. In the overall analysis of mineral elements and ammonia in substrates it is however important to highlight the shortcomings of recalcitrance hence concepts of bioavailability (Myszograj et al. 2018; Thanh et al. 2016). Lignocellulosic material in substrates locks up minerals so that they may not be accessed by microorganisms. Minerals interact among themselves to form non-active, more active or toxic intermediates. Minerals also tend to adsorb to carbonaceous materials which renders the minerals ineffective in participating in microbial pathways. All these issues require further investigation for a better understanding of this topic in AD. Despite these shortcomings, the inferences made based on direct substrate digestion and ICP-OES-based results are quite informative as a starting point. This method though not ideal, sheds some light for AD Practitioners to at least be able to incorporate mineral element effects in biogas digestion rather than ignore them completely at the detriment of running poorly optimized processes or failing to troubleshoot digester poor performances.

Results of the one-way-ANOVA (F = 0.428; P-value = 0.828 and Fcrit = 2.29) where the F < Fcrit indicate that the test is insignificant. The P-value > 0.05 which is the α value at 95% CI, we fail to reject the null hypothesis which stated that there is no difference between the means of the groups (the six substrates’ average mineral element assays). Therefore, the six (6) different avocado oil processing byproducts contain the same mean value of mineral elements assays although observations (Fig. 1) show that the individual mineral element assays in each substrate were fluctuating. Rousell et al. (2018) have developed a decision-making model regarding mineral elements supplementation in digesters taking into consideration an undersupply and oversupply from the substrate itself. The benefits of optimal digester performance by mineral elements supplementation should always be weighed against the risks of exceeding mineral element levels permissible in digestate for its ultimate use as a biofertilizer. Balancing out high and low mineral elements arising in two different substrates through a co-digestion scheme (Habagil et al. 2020) and improving mineral bioavailability by dosing EDTA are additional strategies employed to optimize mineral element levels in digesters.

## Conclusions

It is important to carry out mineral elemental analysis of AD substrates, especially for new and unknown substrates. This analysis helps in explaining substrate digestion behaviour and devising possible optimization strategies for the AD process by adjusting levels of these elements in the digester where necessary. Mineral elements promote microbial growth and activities although this is dependent on concentration and elemental bioavailability. Lower concentrations may lower microbial growth and specific microbial functions while high assays may be toxic to the other microbes. At times, biogas Researchers choose to or not to perform substrate mineral elemental analysis depending on their pre-existing knowledge of the substrate, recommendations from standards adopted in their research and objectives of their studies. South African avocado oil processing byproducts from Westfalia Fruit factories contain different mineral elements in different proportions. Further studies are recommended to confirm and address potential seasonal and operational site-specific variations as well as bioavailability concerns. Going forward, substrate biodegradability evaluation standard protocols could consider incorporating mineral element analysis as a compulsory component of the standard guidelines. Results from these studies are key in developing mineral element testing, supplementation, reduction and detoxification strategies for various biogas digester substrates.

## Data Availability

No datasets were generated or analysed during the current study.

## Code availability

Not available.
